# Development of a novel PRO instrument for use in familial chylomicronemia syndrome

**DOI:** 10.1186/s41687-021-00347-5

**Published:** 2021-08-11

**Authors:** David Davidson, Christina Slota, Montserrat Vera-Llonch, T. Michelle Brown, Andrew Hsieh, Sheri Fehnel

**Affiliations:** 1 NorthShore University HealthSystem, 2151 Waukegan Rd, Bannockburn, IL 60015 USA; 2 RTI Health Solutions, 3040 East Cornwallis Road, Research Triangle Park, NC 27709 USA; 3 Ionis Pharmaceuticals, 2855 Gazelle Court, Carlsbad, CA 92010 USA

**Keywords:** Familial chylomicronemia syndrome, Patient-reported outcomes, PRO measure, FCS Symptoms and Impacts Scale, Clinical outcome assessment, COA

## Abstract

**Background:**

Familial chylomicronemia syndrome (FCS), a rare genetic disorder characterized by high levels of circulating triglycerides, negatively impacts multiple organs, including the liver and pancreas.

**Objective:**

The objective of this study was to develop and support the content validity of a novel patient-reported outcome (PRO) measure addressing FCS symptoms and impacts. To facilitate use in clinical trials of new treatments, evidence supporting the new measure needed to be consistent with regulatory guidance and requirements.

**Methods:**

A pool of items addressing symptoms and impacts of FCS was initially developed based on data from a large burden-of-illness study with patients with FCS as well as a review of available literature and existing PRO measures. Two rounds of qualitative interviews were conducted with patients with FCS (N = 10) to refine the draft items and support the measure’s content validity. Each interview began with concept elicitation followed by cognitive debriefing of the draft FCS measure.

**Results:**

Patient-reported symptoms and impacts of FCS were generally consistent with those identified by the burden-of-illness study; abdominal pain was particularly prevalent and salient for patients. Suggested changes to the draft item pool were generally minor. Comprehensibility and ease of completion for the final instrument were confirmed during the second set of interviews.

**Conclusion:**

The content validity of the final 17-item FCS Symptoms and Impacts Scale is strongly supported by patient input gathered through both a large burden-of-illness study and qualitative research. To further support use in clinical trials, psychometric evaluation of the measure is underway.

## Background

Familial chylomicronemia syndrome (FCS) is a rare genetic disorder characterized by high levels of circulating triglycerides, which result in negative impacts on multiple organs, including the liver and pancreas [1]. Clinical signs and symptoms of FCS include xanthomas, chronic anemia, and hepatosplenomegaly, and patients with the disorder have reported experiencing abdominal pain and fatigue as well as brain fog, anxiety, and depression [2]. Results of a recent online survey to capture input from patients with the disorder have shown that these symptoms and the acute pancreatitis attacks that are triggered by FCS present major impacts on the lives of patients in terms of both the burden of illness and patients’ quality of life [3].

The use of patient-reported outcome (PRO) measures is common in clinical trials and has been noted to be particularly crucial to trials in rare diseases [4–6]. Although generic PRO measures can address some of the symptoms and impacts that patients experience, there are no existing PRO instruments specifically designed to comprehensively capture the concepts of importance to patients with FCS. In order to capture data essential to the evaluation of new treatments for FCS, development of a novel, disease-specific PRO measure was undertaken.

The United States (US) Food and Drug Administration (FDA) guidance *Patient-Reported Outcome Measures: Use in Medical Product Development to Support Labeling Claims* [7] describes regulatory recommendations and criteria used to review PRO measures proposed by sponsors to support product approval or labeling claims. Central to the recommended development process is the incorporation of input from patients in the target population to ensure the relevance and importance of concepts being addressed in PRO measures. While patient input is most commonly solicited in the context of qualitative research, additional sources of patient-reported data (e.g., patient surveys) can facilitate the identification of important concepts for measurement. The objective of this study was to develop and support the content validity of a novel PRO measure of FCS symptom severity and impacts that could be used in clinical trials to support drug approvals in the US and Europe. The resulting instrument, the FCS Symptoms and Impacts Scale, is described and presented here.

## Materials and methods

After relevant concepts were identified from the literature, the FCS Symptoms and Impacts Scale was developed iteratively to incorporate concepts of importance to patients with FCS and ensure easy comprehension of the measure’s instructions, questions, and response options. While staying true to the FDA PRO guidance, some flexibility was permitted in the concept identification process and qualitative research sample size to address the challenge of developing a new measure for use in a rare disease.

### Development of the initial item pool

Based on a review of the data collected during a large, cross-sectional, burden-of-illness study of adults with FCS [3], a review of the relevant clinical literature [1, 8, 9], and existing measures used in relevant conditions such as pancreatitis, draft items were generated to assess the severity and impact of FCS symptoms. While the FDA PRO guidance references measure development that begins with the elicitation of concepts prior to item development, due to the rarity of FCS, the approach described here was taken so that both elicitation of concepts and debriefing of draft items could occur in each patient interview. In order to capture day-by-day variability, items assessing symptom severity were designed for daily completion using a 24-h recall period; items assessing the impacts of FCS were designed to be administered less frequently and ask participants about “current” impacts. All items were designed to be patient-completed, and multiple items were initially drafted to measure some concepts, such as dietary restrictions, in order to determine the clearest and most appropriate wording.

### Qualitative interview methods

Two rounds of qualitative interviews were undertaken with patients with FCS to elicit symptom and impact concepts of importance to patients and to evaluate and cognitively debrief the draft PRO measure. Patient participants were identified through collaboration with patient advocacy groups and clinicians. Interested individuals were screened by a medical recruiter to confirm eligibility. Eligible patients were those aged 18 years or older with a self-reported, physician-provided diagnosis of FCS. All participants provided informed consent prior to the start of the interviews.

Each interview began with a concept elicitation portion during which open-ended questions were posed to get participants talking about their experiences with symptoms associated with FCS and the impacts of these symptoms. This general discussion was followed by more targeted questions designed to identify the improvements that patients would need to experience to feel that a treatment was working, and to determine the most bothersome symptoms and impacts of FCS.

After discussing their own symptoms and experiences, participants were asked to review the draft PRO items and provide feedback on the instructions, questions, and response options using a think-aloud process as they completed the draft measure. In the first round of interviews, objectives included testing the relevance of the concepts, clarity of the instructions and items, and the comprehensiveness of the draft measure. Any issues identified during this round of interviews were used to inform revisions or modifications made before the second round of interviews.

Modifications to the items were made to incorporate feedback collected from participants during the first round of interviews and to address recommendations from a translatability assessment, an evaluation which flags concepts or terminology that cannot be easily translated across languages or cultures. As detailed in the cognitive debriefing results, all changes or modifications based on the first round of interviews and the translatability assessment were tested during the second round of interviews, and the content validity of the instrument was further evaluated.

## Results

### Development of the draft FCS measure

To begin development of the draft measure, an initial pool of 74 symptom and impact concepts was created. Similar concepts reported within and between the sources were evaluated to determine whether they could be combined; the concept of “abdominal pain,” for example, was determined to include abdominal discomfort, pain, sleep disrupted related to pain, pain at night, and pancreatic pain. For each concept or collapsed concept, the following elements were given particular weight in evaluation, as obtained from the study by Davidson and colleagues [3]: a reporting prevalence of 20% or higher, higher severity ratings (i.e., > 4.5 on a 1 to 7 scale), and more frequent occurrence (e.g., daily or every other day). Taking all of this information into account, the initial draft measure, consisting of 4 symptom items and 10 impact items, was developed. In line with FDA guidance [7], each item was designed to assess a single concept that was identified as important and relevant to the experience of FCS.

### Qualitative interview participants

In total, 10 patients with FCS participated in qualitative research interviews discussing their FCS symptoms and the impacts that FCS had on their lives as well as providing feedback on the draft instrument. Round 1 interviews included 6 patients with FCS (60.0%) and Round 2 interviews included 4 patients (40.0%). Participant characteristics are presented in Table 1.

**Table 1:** Demographic and clinical characteristics of interview participants

Patient characteristic	Total (N = 10)
Sex, n (%)	
Female	7 (70.0)
Male	3 (30.0)
Age in years, mean (range)	53.0 (28–69)
Race, n (%)	
White	10 (100.0)
Education, n (%)	
Some college	2 (20.0)
College degree	3 (30.0)
Some graduate school	3 (30.0)
Professional or advanced degree	2 (20.0)
Body mass index, mean (range)	25.3 (18–34)
FCS confirmed through genetic testing, n (%)	9 (90.0)
Participated in FCS clinical trial within the past 2 years, n (%)	6 (60.0)
Still taking study drug, n (%)	4 (40.0)
Diagnosed with diabetes, n (%)	6 (60.0)

*FCS* familial chylomicronemia syndrome

### Concept elicitation results: symptoms of FCS

The mean age of participants at the time they received their FCS diagnosis was 35 years, although the range spanned from infancy to 65 years of age. When asked about the symptoms they had experienced that led to their diagnosis, most participants described experiencing abdominal pain (n = 7); 4 participants reported that their FCS was first identified through abnormal bloodwork.

Interview participants identified abdominal pain (100.0%; n = 10), loose bowel movements or diarrhea (80.0%; n = 8), brain fog (60.0%; n = 6), and fatigue (60.0%; n = 6) as being the most common symptoms of FCS that they continued to experience (Table 2). Outside of the pain experienced during a pancreatitis attack, most participants (70.0%; n = 7) reported experiencing low-grade abdominal pain (i.e., a score of 1–4 on a 0-to-10 numerical rating scale [NRS]) on a daily basis.

- I think that I probably always have, like, a little bit of [abdominal] discomfort. So, I would say, like, a 1 or a 2. I don’t think that I really have any days that it’s just totally pain free.
- Pain in my left side most all the time… I would say at least 3 days a week, the pain. It goes to your back.

**Table 2:** Symptoms and impacts of FCS reported by interview participants

Concept	Interview number										Total, n (%) (N = 10)
	1	2	3	4	5	6	7	8	9	10	
Symptoms											
*Abdominal pain*	**✓**	**✓**	**✓**	**✓**	**✓**	**✓**	**✓**	**✓**	**✓**	**✓**	10 (100.0)
*Loose bowel movements/diarrhea*	**✓**	**✓**	**✓**	**✓**	**✓**	**✓**		**✓**		**✓**	8 (80.0)
*Difficulty thinking/mental fatigue/brain fog*	**✓**		**✓**	**✓**	**✓**	**✓**		**✓**			6 (60.0)
*Fatigue (physical)*	**✓**		**✓**	**✓**	**✓**	**✓**			**✓**		6 (60.0)
Blurred vision	**✓**	**✓**	**✓**	**✓**	**✓**						5 (50.0)
Bloating		**✓**	**✓**	**✓**	**✓**	**✓**					5 (50.0)
Difficulty remembering words or names	**✓**	**✓**	**✓**	**✓**				**✓**			5 (50.0)
Xanthomas	**✓**		**✓**		**✓**	**✓**					4 (40.0)
Poor appetite	**✓**	**✓**		**✓**	**✓**						4 (40.0)
Nausea		**✓**			**✓**			**✓**		**✓**	4 (40.0)
Pain (not abdominal)	**✓**		**✓**	**✓**						**✓**	4 (40.0)
Difficulty concentrating		**✓**						**✓**	**✓**		3 (30.0)
Vomiting				**✓**	**✓**			**✓**			3 (30.0)
Weight loss	**✓**				**✓**						2 (20.0)
Indigestion			**✓**			**✓**					2 (20.0)
Muscle weakness			**✓**			**✓**					2 (20.0)
Impacts											
Daily functioning	**✓**	**✓**	**✓**	**✓**	**✓**	**✓**	**✓**	**✓**	**✓**	**✓**	10 (100.0)
Social functioning	**✓**	**✓**	**✓**	**✓**	**✓**	**✓**	**✓**	**✓**	**✓**	**✓**	10 (100.0)
Mental/emotional well-being	**✓**	**✓**	**✓**	**✓**	**✓**	**✓**	**✓**	**✓**	**✓**	**✓**	10 (100.0)
Ability to work/volunteer	**✓**	**✓**	**✓**	**✓**	**✓**	**✓**		**✓**	**✓**	**✓**	9 (90.0)
Financial	**✓**				**✓**	**✓**		**✓**	**✓**		5 (50.0)

Symptoms in *italics* are included in the final FCS Symptoms and Impacts Scale

*FCS* familial chylomicronemia syndrome

The same percentage of participants (70.0%; n = 7) further reported that the acute abdominal pain that occurred during acute pancreatitis attacks was the single most bothersome symptom. Descriptions of the abdominal pain they experienced emphasized both the severity and location of the pain and the impact that this pain had on patients.

- When it becomes acute pancreatitis, you are vomiting bile, you are in extraordinary pain here [pointing at abdomen], all the way around to your back, and you’re just doubled over.
- I can’t even hardly touch my stomach [it’s so painful].

The importance of addressing pancreatitis attacks and the corresponding abdominal pain was further reflected in statements by many participants who noted that prevention of such attacks would be a key target for any successful treatment of FCS.

- If I could start to eat more like what I want to eat and not come down with an attack or a problem, that would be nice.
- [With a new medication] I would’ve wanted the energy definitely and then to take away the fear of getting pancreatitis.

### Concept elicitation results: impacts of FCS

During the open-ended portion of the interview, patients also elaborated on the impacts they experienced related to FCS and their FCS symptoms, describing limitations in their daily functioning (100.0%; n = 10); social functioning (100.0%; n = 10); and mental or emotional well-being (100.0%; n = 10), including worry, anxiety, and depression (Table 2).

- I push through a lot of daily activities to be able to work. It’s a struggle. The fatigue. Just no motivation, no energy, because the energy’s not there.
- I’m really going to have to push myself to do those things that normally would be so easy to do, like go out for a run or go out for a walk or take a shower or, um, get dressed out of my pajamas.
- I avoid making plans with other people because I don’t know if I’ll feel well.
- I hated, you know, leaving my house ever to go out of town or do anything because I never knew when I was going to get sick.
- Mentally it’s not an easy thing. Living with FCS is like having a ticking bomb in your pocket and not knowing when it’s going to go off. There’s absolutely no rhyme or reason… out of the blue, you get pancreatitis, vomit, and diarrhea.
- There was this constant fear of, like, am I going to wake up tomorrow and have to, you know, go to the emergency room. I couldn’t relax.

In addition, 90.0% of participants (n = 9) also described impacts in their ability to work or volunteer and half of the participants (50.0%; n = 5) indicated that their FCS had caused financial impacts (Table 2).

- I finally had to give up my job at the end of August this year, and that was hard.
- Whenever you’re sick and you’re working… I mean, people just don’t understand. And so, if they’re going to have to lay people off, you’re one of the first people. This is the third job I’ve been laid off from.
- Like, we’ve lost our home. We’ve gone bankrupt. I’ve had $100,000 medical bills with insurance.
- With the hospital stays and then not having adequate insurance because we couldn’t afford good insurance… just all the hospital bills coming in and the hospitals calling every day because you can’t make a payment or you can’t, you know. It’s just financially draining.

### Cognitive debriefing results: symptom section of the FCS symptoms and impacts scale

In both Round 1 and Round 2 interviews, patients with FCS were asked to review 4 items addressing symptoms of FCS and to provide feedback on a 0-to-10 NRS for use in responding to symptom items. The NRS ranged from 0 (No [symptom]) to 10 (Worst possible [symptom]). In both rounds of interviews, all participants indicated that the NRS was appropriate and easy to use.

The four symptoms included in the Round 1 draft measure were abdominal pain, physical fatigue, abdominal bloating, and physical weakness. While the concepts of abdominal pain and physical fatigue were determined to be clear, participants in Round 1 indicated that the concept of abdominal bloating was not particularly relevant to their FCS, as bloating could be caused by other factors, such as traveling or food choices; additionally, the concept of physical weakness was considered to largely overlap with the concept of physical fatigue, which participants considered to be more meaningful. As such, the items assessing abdominal bloating and physical weakness were omitted after Round 1 interviews.

Based on a number of reports from participants during the concept elicitation portion of Round 1 interviews, two new FCS symptoms were identified: difficulty thinking (“brain fog”) and diarrhea. Items assessing these concepts were added after Round 1 and tested with participants in Round 2 interviews. To address feedback from the translatability assessment, the difficulty thinking item was tested without the term “brain fog” with patients in Round 2 interviews. Participants with FCS confirmed the relevance of the concept and found the item to be clear and easy to answer, supporting its inclusion in the final instrument. An item assessing diarrhea was also added to the draft instrument based on results of the concept elicitation portion of Round 1 interviews. Participants in Round 2 indicated that this was an important symptom to assess and that the item was clear and easy to answer.

On the basis of this patient input, the final FCS Symptoms and Impacts Scale includes 4 key symptom items: abdominal pain, physical fatigue, difficulty thinking, and diarrhea.

### Cognitive debriefing results: impacts section of the FCS symptoms and impacts scale

Items addressing the impacts of FCS were evaluated in two rounds of cognitive debriefing interviews, with 10 impact items tested in Round 1 interviews and 17 impact items tested in Round 2 interviews. In each round, patients with FCS were asked to review the impact items along with draft instructions and a 5-point verbal response scale (VRS) for use in responding to impact items. Instructions invited participants to “think about how your FCS is currently affecting your life,” and all participants in both rounds of interviews found the instructions to be clear and easy to understand. Similarly, all 10 participants indicated that the VRS was easy to use in completing the questions and that the answer choices presented (Never, Rarely, Sometimes, Often, and Always) were appropriate and distinct. One minor wording change was made to the VRS following a translatability assessment conducted after Round 1 (i.e., the response ‘Usually’ was changed to ‘Often’); this change was tested with participants during Round 2 interviews and confirmed to be appropriate.

The 10 draft impact items tested in Round 1 included worry about severe pain, worry about pancreatitis attacks, difficulty sleeping, impacts on social activities, dietary restrictions, worry about future health issues, and limitations on physical activity. The items assessing worry about severe pain and worry about pancreatitis attacks were reported by participants with FCS to be redundant, as severe pain was only experienced during a pancreatitis attack. The item about severe pain was therefore removed following Round 1 interviews; the item on worry about pancreatitis attacks was retained.

The item assessing social activities (“I feel anxious in social situations involving food”) was shown to be highly relevant to participants, who found the item easy to understand and answer and further elaborated on their experience by noting that they often avoided certain social situations that involved food. To reflect this experience and capture information related to it, a separate item was added following Round 1 interviews to assess avoidance of social situations involving food. The new item was tested in Round 2 and its importance and comprehensibility were confirmed.

With regard to dietary restrictions, items assessing worry about eating food prepared by someone else and worry about going over dietary fat limits were observed to be meaningful to patients with FCS. Participants reported that both of these items were clear, relevant, and easy to answer. Both items were retained with no changes.

The item addressing worry about future health issues was reported to reflect an important impact of FCS. Interview participants across both rounds found the item to be easy to understand and answer. It was retained in the final instrument with no changes.

The draft item addressing limitations in physical activity was confusing for participants in Round 1. Participants felt that the wording of the item (“I limit my physical activity”) did not accurately capture their experience. An alternative phrasing was proposed (“I am less physically active than I would like to be”) and tested in Round 2 interviews. Participants in the second round of interviews found the revised item to be relevant and easy to answer; the revised item was therefore retained in the final instrument.

Participant feedback in Round 1 identified three impact concepts that were initially included in the draft measure as being not relevant to FCS or as unimportant. As a result of this feedback, difficulty sleeping, having trouble following dietary restrictions, and fasting to avoid symptoms of FCS were all omitted from the draft measure following Round 1 interviews. Further, new concepts that were introduced during the concept elicitation portion of interviews were added to the draft measure. Due to the number of reports from participants during Round 1 interviews, 6 new impacts of FCS were identified: avoiding social plans, worry about being a burden, depression, worry about judgement from others, financial difficulties, and impacts on productivity. Items assessing these concepts were added to the draft measure after Round 1 and tested with participants in Round 2 interviews. No new concepts were reported in the Round 2 interviews, indicating that concept saturation had been met. All of these concepts tested well in the second round of interviews and were retained for the final FCS Symptoms and Impacts Scale.

In total, the final FCS Symptoms and Impacts Scale includes 13 impacts: worry about pancreatitis attacks, avoiding making social plans, feeling anxious in social situations involving food, avoiding social situations involving food, worry about eating food prepared by others, worry about exceeding dietary fat limits, worry about future health, worry about being a burden, being less physically active, feeling sad or depressed, feeling judged by others, worry about finances, and being less productive.

## Discussion

In the absence of a disease-specific PRO instrument for FCS, development of a novel measure was initiated using data from a large, patient-completed, burden-of-illness study and a review of the available literature. After these sources were used to identify concepts of importance and establish an early framework, qualitative research was conducted with patients to confirm and identify any additional symptoms and impacts of FCS of key importance and refine the initial item pool to yield an FCS-specific PRO measure appropriate for use in clinical trials.

In identifying a comprehensive set of 74 potentially unique symptoms and impacts of FCS, the significant burden of FCS was confirmed, both in terms of the breadth and the severity of patient experiences. Furthermore, the variability and unpredictability of the disease was described as troublesome to patients, reflecting not only the complexity of the disease presentation and course but also delays in disease diagnosis, proper treatment, and the measurement of treatment benefit in clinical trials. Notably, the most severe, bothersome, and impactful symptom—acute pancreatitis—is episodic; the intermittent presentation of this symptom exemplifies the challenges posed in attempting to capture the potential benefits of treatment for FCS. That said, the occurrence of long-term pancreatic dysfunction or chronic pancreatitis in FCS has been suggested, resulting in more frequent or consistently experienced symptoms, including abdominal pain and diarrhea [1, 10]. Notably, 7 of the 10 patients in this study reported experiencing low-grade abdominal pain on a daily basis. Impacts, too, reflect both the unpredictability and chronic course of the disease and the ways in which patients attempt to manage their FCS and its symptoms. In a recent study, patients with FCS reported both abdominal (“belly”) pain and pain interference considered clinically meaningful based on comparisons to general population norms [11, 12]. Similar findings were also reported for cognition impacts [11]. In the current study, worry or anxiety was a common theme when patients described ongoing impacts of FCS. For example, patients reported worrying about the ramifications of exceeding their daily fat limit, about the acute medical issues that can occur without warning, and about hospitalization and the corresponding financial impacts.

To our knowledge, this research is the only published qualitative research study conducted in patients with FCS. Despite the difficulties described and the variability inherent in the disease, interviews with patients with FCS revealed a set of core symptoms and impacts that participants felt were distinct from one another and essential for capturing the benefit of a treatment for FCS. These concepts represent the foundation for the FCS Symptoms and Impacts Scale and cognitive debriefing results indicate that patients consider the content of the measure to be relevant and important and the response options to be clear, appropriate, and easy to use.

Limitations of the current study include the small sample size and the fact that findings may not be representative of the larger population of patients with FCS. This weakness is in part inevitable due to the rarity of the disease but may be mediated by the fact that most patients reported receiving genetic confirmation of their FCS (underscoring the credibility of the participants and the reliability of the feedback they provided) and by the fact that concept saturation was reached, indicating that all significant concepts were identified despite the small sample size. One further factor to note when considering the sample is the fact that 6 interview participants (60.0%) had previous experience participating in a clinical trial for an FCS treatment. While this experience may present a limitation by potentially reducing the variety or severity of participant’s current symptoms, it also serves as a research strength, as it allows a portion of the sample to speak more knowledgeably about the impact and importance of improvements or changes in symptoms and impacts.

## Conclusions

This study was undertaken to address an unmet need for a disease-specific, fit-for-purpose PRO instrument capable of demonstrating improvements in the core symptoms and impacts of FCS. In alignment with best practices for PRO measure development outlined by the FDA, concepts of importance to patients were identified from the results of a large burden-of-illness study of adults with FCS and a review of the relevant clinical literature, and feedback on the content and format of the measure was collected through two rounds of qualitative interviews with patients with FCS. The resulting instrument, the FCS Symptoms and Impacts Scale, is a 17-item PRO measure of the 4 most important symptoms and 13 most important impacts in FCS; the measure was developed with input from patients with FCS and has demonstrated strong content validity. Qualitative interviews with patients indicate that the FCS Symptoms and Impacts Scale has strong potential to show the benefit of FCS treatments when incorporated into clinical trials. Research is planned to evaluate the psychometric properties of the FCS Symptoms and Impacts Scale as well as its ability to demonstrate drug benefit in this patient population and support potential labeling claims.

## Data Availability

Not applicable.

## Abbreviations

FCS: Familial chylomicronemia syndrome
FDA: US Food and Drug Administration
NRS: Numeric rating scale
PRO: Patient-reported outcome
US: United States
VRS: Verbal response scale
